# Integrative taxonomy of the genus *Nesticus* in central Japan, with the description of a new species and redescriptions of *N. echigonus* and *N. gondai* (Araneae, Nesticidae)

**DOI:** 10.3897/zookeys.1285.202554

**Published:** 2026-07-17

**Authors:** Toshimichi Nagai, Francesco Ballarin, Katsuyuki Eguchi

**Affiliations:** 1 Systematic Zoology Laboratory, Department of Biological Sciences, Tokyo Metropolitan University, 1-1 Minami-Osawa, Hachioji-shi, 192-0397, Tokyo, Japan Systematic Zoology Laboratory, Department of Biological Sciences, Tokyo Metropolitan University Tokyo Japan; 2 Department of Zoology, Museo di Storia Naturale of Verona, Lungadige Porta Vittoria, 9, I-37129 Verona, Italy Department of International Health and Medical Anthropology, Institute of Tropical Medicine, Nagasaki University Nagasaki Japan; 3 Department of International Health and Medical Anthropology, Institute of Tropical Medicine, Nagasaki University, 1-12-4 Sakamoto, Nagasaki-shi, 852-8523, Nagasaki, Japan Department of Zoology, Museo di Storia Naturale of Verona Verona Italy

**Keywords:** COI, morphology, phylogeny, spider, troglophiles

## Abstract

Species of the genus *Nesticus* (Araneae, Nesticidae) in central Japan are revised based on morphological and molecular analyses of specimens collected from Niigata and Gunma Prefectures. Among them, a putative undescribed species was discovered having clearly distinguishable morphological characters from other known species in the same area. A phylogenetic analysis based on the mitochondrial *COI* gene was carried out using a maximum-likelihood method to confirm its distinctiveness. The new species, *Nesticus yamabushi***sp. nov**., is described based on specimens of both sexes. Furthermore, we provide detailed redescriptions of two other closely related species, *N. echigonus* and *N. gondai*, to address the lack of information in their original descriptions and facilitate future identifications. This study enhances our understanding of the fauna and diversity of the genus *Nesticus* in central Japan and provides a foundation for future taxonomic research.

## Introduction

Nesticidae Simon, 1894 (Arachnida, Araneae) is a relatively small family of spiders currently comprising 16 genera and 281 species with a nearly worldwide distribution (World Spider Catalog 2026). Within Nesticidae, the genus *Nesticus* Thorell, 1869 is the most species-rich with 119 valid species and subspecies distributed across the Palaearctic, Nearctic, and Neotropical regions. Species of this genus are typically troglophilic or troglobitic, occurring in caves, artificial tunnels, mountain scree, shaded slopes, and other similarly moist habitats with relatively stable temperatures (Suzuki and Ballarin 2020). Recent phylogenetic studies have revealed that *Nesticus* is not monophyletic, prompting an ongoing partial taxonomic revision of the genus (Ribera and Dimitrov 2023). Accordingly, numerous species have been progressively transferred to different genera over time (World Spider Catalog 2026). Nevertheless, the revision of the genus is still far from complete, as previous studies have so far focused mainly on European species, whereas the Asian taxa have been only partially revised (Lin et al. 2016) and American ones remain largely unrevised.

In Japan, 44 species and six subspecies of *Nesticus* sensu lato have been described to date (Japan Spider Catalog 2026), accounting for approximately 35% of the genus’s current total diversity worldwide. This makes the Japanese Archipelago a global hotspot for *Nesticus* and Nesticidae in general. The genus is widely distributed throughout mainland Japan but is absent from the Ryukyu Archipelago. It exhibits high levels of endemism, with numerous species restricted to small areas or even single caves. However, it is likely that its diversity is still underestimated and numerous species remain undiscovered (Ballarin and Eguchi 2023), partly due to the lack of detailed information on known species.

Most of the Japanese *Nesticus* species were described in the 1970s and 1980s (World Spider Catalog 2026). Their descriptions were based solely on morphological characters, and several species still lack detailed descriptions and modern illustrations of their diagnostic characters. In addition, the use of integrative approaches combining both morphological and molecular data have only recently begun in Japan for nesticid species delimitation, and these data are still limited (Suzuki and Ballarin 2020; Ballarin and Eguchi 2023). Accordingly, more comprehensive studies are necessary to properly understand the true diversity of the Japanese nesticid fauna.

During field surveys in central Japan, specifically in Niigata and Gunma Prefectures, we collected several *Nesticus* specimens. A detailed examination of these specimens revealed a putative undescribed species. This examination also highlighted the need for a taxonomic re-evaluation of known related species from the same geographic area to facilitate a more rigorous comparison. In this study, we aim to confirm the taxonomic distinctness of the putative undescribed species using an integrative approach combining morphological and molecular data. Furthermore, we redescribe two other closely related species, *N. echigonus* and *N. gondai*, whose original descriptions are taxonomically ambiguous due to outdated terminology, lack a description of the vulva, and are accompanied only by simplified illustrations.

## Materials and methods

### Field collection and morphological study

Spider specimens were hand-collected in central Japan from leaf litter, culverts, and gaps between stones near mountain streams. Samples were stored in 70% and 99% ethanol for morphological and molecular studies, respectively. Photographs of microhabitats or live specimens were taken with an Olympus Tough TG-5 camera or an iPhone SE (2^nd^ generation). Live individuals were photographed indoors using a white plastic board as a background. Subadult specimens were collected in the field and reared to maturity at room temperature over a period of several days to months in the laboratory of Tokyo Metropolitan University (TMU), Japan. During this period, specimens were kept in plastic cases measuring 11.5 × 16 × 11.5 cm to maintain a high relative humidity and were fed small insects, such as cockroach nymphs collected outdoors. All experiments were conducted at the same institute.

Specimens were examined under a Nikon SMZ1270 stereomicroscope. Photographs and measurements of the habitus and genitalia were taken under a Nikon AZ 100 stereomicroscope with a Canon EOS R10 camera mounted on it. Photographs were subsequently stacked using Helicon Focus v. 7.6.6 software. Male palps and epigynes were dissected using fine forceps and a dissecting needle for better observation and photography. The left male palp was illustrated. Epigynes were cleared by soaking in a hot 20% KOH solution for several minutes and stained with methylene blue to better visualize their transparent internal structures. Leg measurements are reported as follows: total (femur, patella, tibia, metatarsus, tarsus). For previously described species, the redescription and illustrations of the male were based on the holotype, while those of the female are based on a fresh voucher specimen compared with the paratype, to avoid damaging the type specimen during the examination of the vulva.

Abbreviations used in the text and figures are as follows, mostly following Suzuki and Ballarin (2020):

**AME** = anterior median eyes; **ALE** = anterior lateral eyes; **C** = conductor complex; **Co** = copulatory opening; **Da1-5** = dorsal apophyses of the paracymbium; **Di** = Distal apophysis of paracymbium; **E** = embolus; **Id + Fd** = insemination and fertilization ducts; **Ma** = median apophysis; **Ms** = median septum; **P** = paracymbium; **Pc1-4** = processes of the conductor complex; **PME** = posterior median eyes; **PLE** = posterior lateral eyes; **PLS** = posterior lateral spinnerets; **Rx** = radix; **S** = spermatheca; **Sc** = scape; **T** = tegulum; **Ta** = tegular apophysis; **Va** = ventral apophysis of the paracymbium; **VpA** = anterior vulval pocket; **VpM** = medial vulval pocket.

Specimens examined in this study are deposited in the collections of the following institutions:

**OMNH** = Osaka Museum of Natural History, Osaka, Japan; **MNHH** = Museum of Nature and Human Activities, Hyogo, Japan; **MNSK** = Echigo-Matsunoyama Museum of Natural Science “Kyororo”, Niigata, Japan; **NSMT** = National Museum of Nature and Science, Tsukuba, Japan; **TNPC** = Toshimichi Nagai personal collection.

### Molecular analysis

For each individual, total genomic DNA was extracted from one or two leg segments, or from an entire leg depending on the specimen size, using a Chelex-TE-ProK protocol (Ballarin and Eguchi 2023) with an incubation time of 12 h. Polymerase chain reaction (PCR) was performed in a MiniAmp Thermal Cycler (Thermo Fisher Scientific, Waltham, Massachusetts, USA) in a 10.5 µL reaction volume containing 5 µL of KOD One® PCR Master Mix (Toyobo, Osaka, Japan; KMM-201), 3.9 µL of distilled water (DW), 0.3 µL each of forward and reverse primers (10 pmol/µL), and 1.0 µL of DNA template. Two primer pairs were used to amplify two partial fragments of the mitochondrial cytochrome c oxidase subunit I (*COI*) gene: LCO1490 (forward) 5’-GGTCAACAAATCATCATAAAGATATTGG-3’ (Folmer et al. 1994) with CHR2 (reverse) 5’-GGATGGCCAAAAAATCAAAATAAATG-3’ (Barrett and Hebert 2005) and C1-J-2183 (forward) 5’-CAACATTTATTTTGATTTTTTGG-3’ (Simon et al. 1994) with C1-N-2776 (reverse) 5’-GGATAATCAGAATATCGTCGAGG-3’ (Hedin and Maddison 2001). The PCR protocol was as follows: initial denaturation at 94 °C for 2 min; 40 cycles of 98 °C for 10 s, 50 °C for 5 s (annealing step), and 68 °C for 1 s, and a final extension at 68 °C for 7 min. The quality of each PCR product was checked by electrophoresis on a 2.0% agarose gel in 1× TAE at 100 V for 11 min. The PCR products were purified using ExoSAP-IT (Thermo Fisher Scientific) (Bell 2008) and sent to Azenta (Tokyo, Japan) for sequencing. The resulting sequences were visually inspected and translated into amino acid sequences with ChromasPro v. 2.1.8 (http://technelysium.com.au/ChromasPro.html) to check for stop codons and potential errors and subsequently aligned using Clustal X v. 2.1 (http://www.clustal.org/). The two gene fragments were concatenated using MEGA 12 (Kumar et al. 2024). The final concatenated alignment was 1,275 base pairs (bp) in length.

The phylogenetic tree was reconstructed using the maximum-likelihood (ML) method. To improve the stability of the tree topology by enriching the taxon sampling of the genus, *Nesticus shinkaii* Yaginuma, 1979 from the close geographic area was also included in the genetic analysis. However, it was omitted from the morphological comparisons due to its distinct morphological differences from the examined taxa. Two species, *Nesticus cellulanus* (Clerck, 1757) and *Kryptonesticus eremita* (Simon, 1880), were selected as outgroups. *Nesticus cellulanus* was included as it is the type species of the genus, while *K. eremita* represents a distinct genus that is relatively closely related to the Asian lineage (Ribera and Dimitrov 2023). The ML analysis was performed in IQ-TREE v. 3.0.1 (Wong et al. 2025) with 1,000 bootstrap replicates under the HKY+F+I nucleotide substitution model automatically selected by the software, with all other parameters kept at their default settings. The resulting phylogenetic tree was visualized and edited using iTOL v. 7 (Letunic and Bork 2024; https://itol.embl.de/).

A pairwise-distance analysis to measure genetic distances between species was conducted using MEGA 12 with 1,000 bootstrap replicates.

The list of specimens used in these analyses and their corresponding GenBank accession numbers are reported in Table 1.

**Table 1.** List of specimens used in the analysis, related specimen ID, GenBank accession code, specific name, locality of collection, and origin of data.

**ID**	**Species**	**GenBank**	**Locality**	**Citation**
29	*Nesticus yamabushi***sp. nov**.	PZ567708	Japan, Niigata, Tokamachi city, Amamizu park (天水公園)	This work
45	*Nesticus yamabushi***sp. nov**.	PZ567707	Japan, Niigata, Tsunan town, Mt. Yamabushiyama (山伏山)	This work
105	*Nesticus yamabushi***sp. nov**.	PZ567711	Japan, Niigata, Tsunan town, Mt. Yamabushiyama (山伏山)	This work
111	*Nesticus yamabushi***sp. nov**.	PZ567710	Japan, Niigata, Tsunan town, Mt. Yamabushiyama (山伏山)	This work
113	*Nesticus yamabushi***sp. nov**.	PZ567709	Japan, Niigata, Tsunan town, Mt. Yamabushiyama (山伏山)	This work
Cy092	*Nesticus yamabushi***sp. nov**.	PZ567713	Japan, Niigata, Tsunan town, Mt. Yamabushiyama (山伏山)	This work
Cy091	*Nesticus echigonus*	PZ567714	Japan, Niigata, Uonuma city, Mt. Asakusadake (浅草岳)	This work
Cy191	*Nesticus gondai*	MT524191	Japan, Gunma, Matsuida town, Kirizumi-gawa river valley, near Kirizumi onsen	(Suzuki & Ballarin, 2020)
94	*Nesticus shinkaii*	PZ567712	Japan, Tokyo, Akiruno city, Yozawa, the 1^st^ cave on opposite shore of the Mitsugo cave (三ツ合対岸の穴第1洞)	This work
Cy189	*Nesticus shinkaii*	MT524192	Japan, Tokyo, Akiruno city, Yozawa, Otake cave (大岳鍾乳洞)	(Suzuki & Ballarin, 2020)
Z244	*Nesticus cellulanus*	MG201044.1	Bulgaria, Shumen Province, Shumen Plateau Nature Park, Zandara cave	(Ballarin & Li, 2018)
K211	*Kryptonesticus eremita*	MK860156.1	Grotta di Ponte Subiolo, Mori, Veneto, Italy	(Ballarin, 2020)

## Results

### Taxonomic account

**Family Nesticidae Simon, 1894**

#### Genus Nesticus

Taxon classificationAnimaliaAraneaeNesticidae

Thorell, 1869

2F675A85-A798-5B8C-97FF-E51020BB396E

##### Type species.

*Araneus cellulanus* Clerck, 1757 from Sweden.

#### Nesticus yamabushi sp. nov.

Taxon classificationAnimaliaAraneaeNesticidae

5F04D141-9EC3-5FFF-A12B-35CBC21B38C7

https://zoobank.org/890DD371-F679-4B1E-A3C4-10F6557A24D8

**Figure 1.**
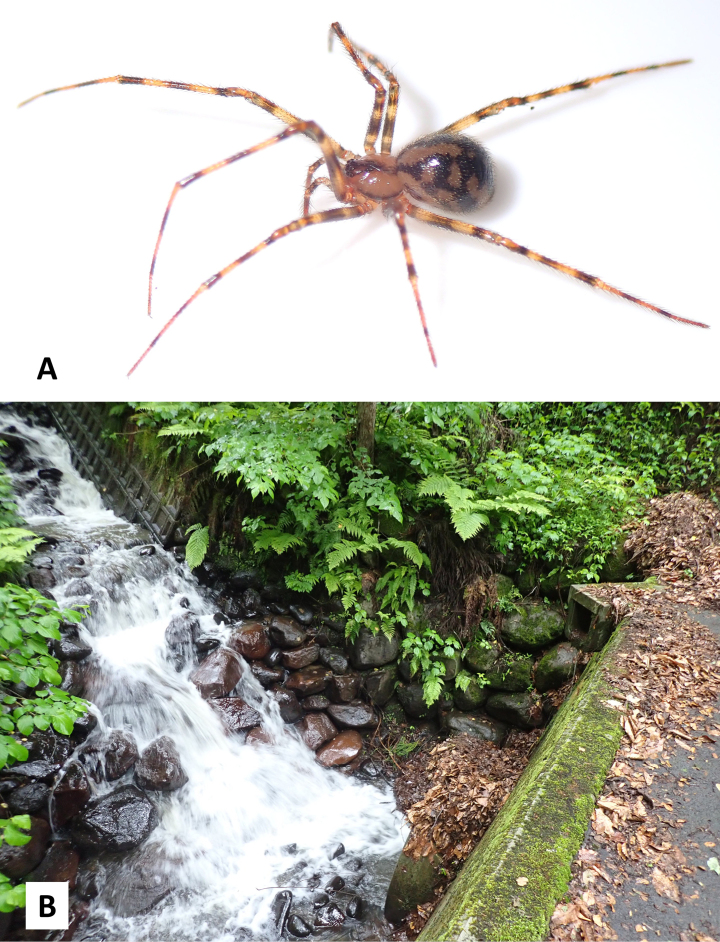
*Nesticus yamabushi* sp. nov. and habitat. **A**. Living specimen; **B**. Example of habitat where the species was collected.

**Figure 2.**
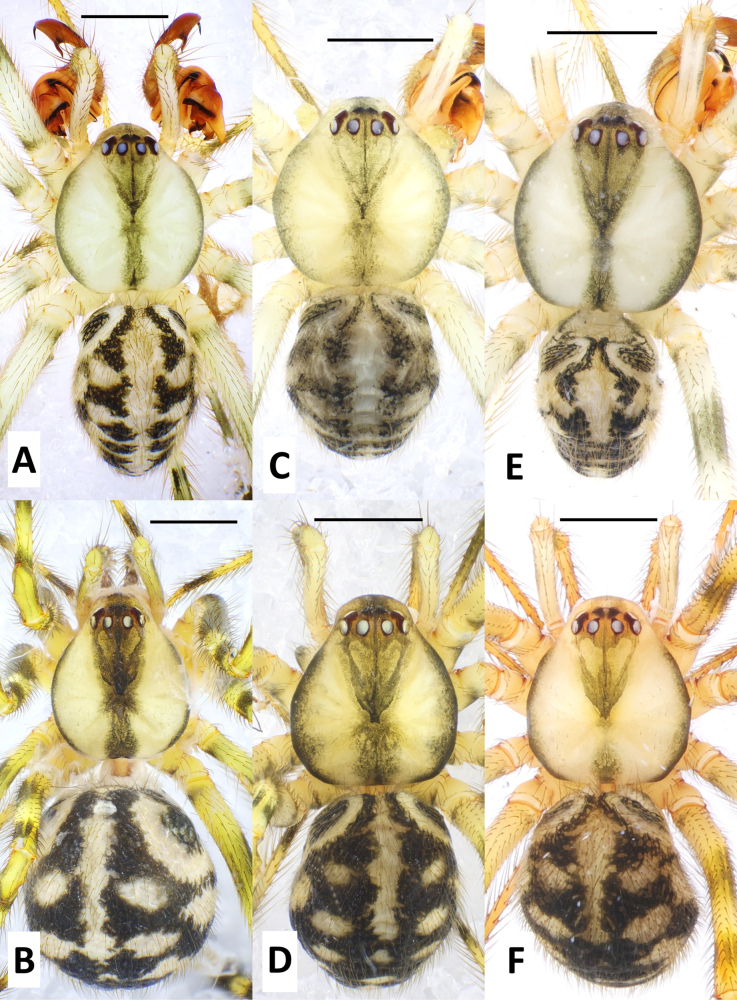
Habitus and dorsal pattern of *Nesticus* species discussed in this work. **A**. Male of *N. yamabushi* sp. nov.; **B**. Same, female; **C**. Male of *N. echigonus*; **D**. Same, female; **E**. Male of *N. gondai*; **F**. Same, female. Scale bars: 1.0 mm.

**Figure 3.**
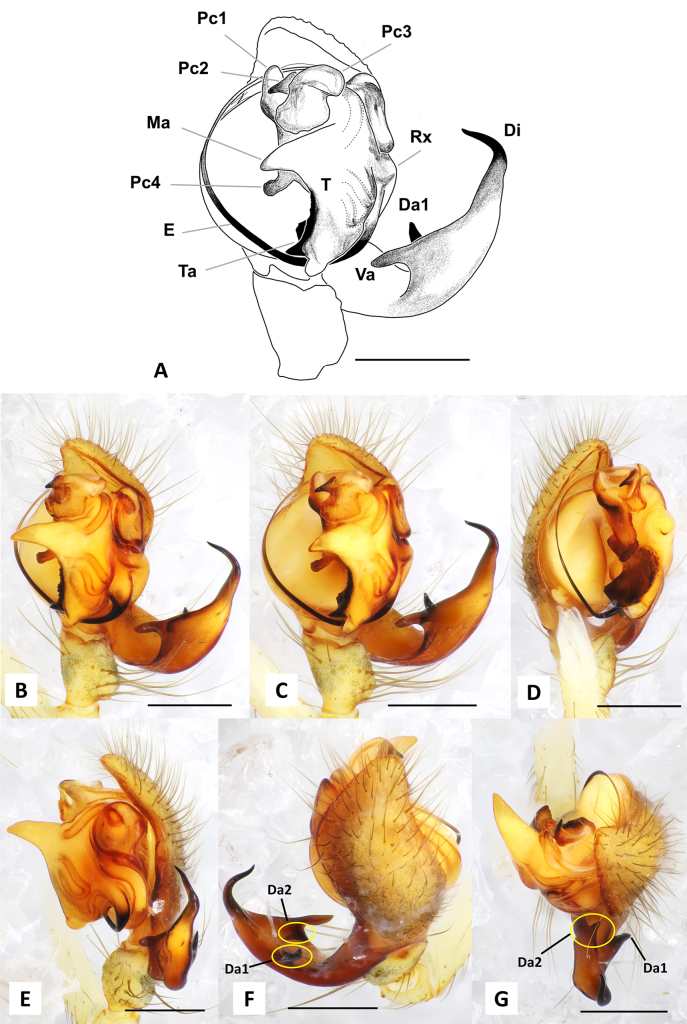
*Nesticus yamabushi* sp. nov., male genitalia of the specimen NSMT-Ar 27107. **A**. Genital sketch, left palp, ventral; **B**. Left palp, ventral; **C**. Same, ventro-retrolateral; **D**. Same, medial. **E**. Same, distal; **F**. Same, dorsal; **G**. Same, anterior. Abbreviations: Da1–2 = dorsal apophyses of paracymbium, Di = distal apophysis of paracymbium, E = embolus, Ma = median apophysis, Pc1–4 = processes of conductor complex, Rx = radix, T = tegulum, Ta = tegular apophysis, Va = ventral apophysis of paracymbium. Scale bars: 0.5 mm.

**Figure 4.**
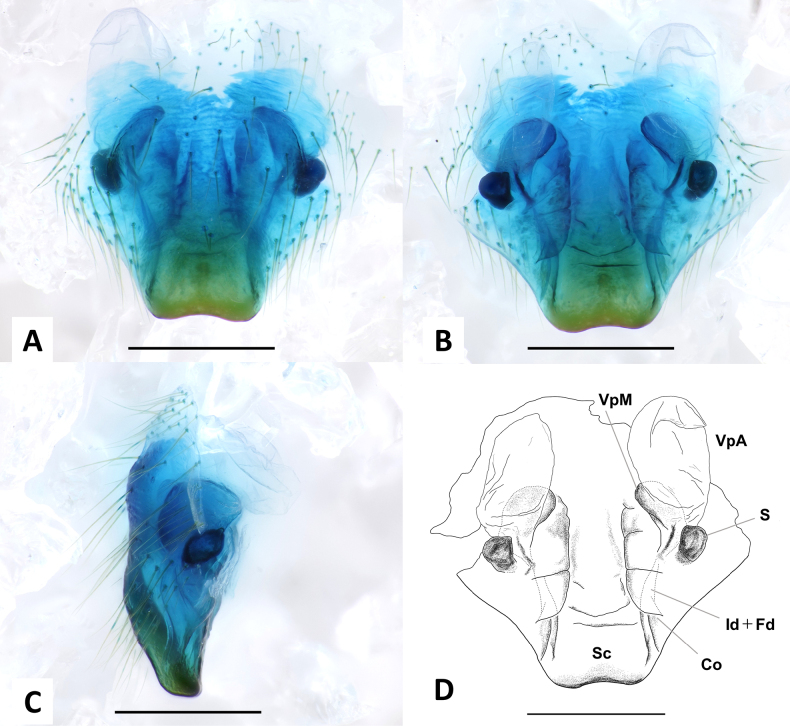
*Nesticus yamabushi* sp. nov. female genitalia. **A**. Epigyne, ventral; **B**. Vulva, dorsal; **C**. Epigyne, lateral; **D**. Genital sketch, vulva, dorsal. Abbreviations: Co = copulatory opening, Id+Fd = insemination + fertilization ducts, S = spermatheca, Sc = scape, VpA = anterior vulval pocket, VpM = medial vulval pocket. Scale bars: 0.5 mm.

Figs 1A, 2A, 2B, 3A–G, 4A–D

##### Japanese name.

Yamabushi-Horahimegumo ヤマブシホラヒメグモ.

##### Materials examined.

• ♂ ***Holotype***: Japan: Honshu Island, Niigata Pref.: Uonuma City, Tsunan Town, Mt. Yamabushiyama, (36.9998°N, 138.5904°E), 306 m elev., 11 June 2022, T. Nagai leg., gaps between stones near mountain stream. (NSMT-Ar 27107).

***Paratypes***: • 1♂, same locality as holotype, 14. Jun. 2022, T. Nagai leg., gaps between stones near mountain stream (NSMT-Ar 27108) • 2♀, same locality and collection date as holotype, T. Nagai leg., gaps between stones near mountain stream (NSMT-Ar 27109) • 1♀, Tokamachi City, Amamizukoshi, bank of Koedougawa river (37.02374°N, 138.350°E), 566 m elev., 02 August 2017, Y. Suzuki leg. under stones near mountain stream. (NSMT-Ar 27110) • 1♂, Tokamachi City, Amamizu Park (37.0508°N, 138.5855°E), 446 m elev., 03 November 2020, mature on 11 January 2021, T. Nagai leg., gaps between stones in park (OMNH) • 1♀, same locality, collection date, and environment, T. Nagai leg. (OMNH) • 1♂, Uonuma City, Tsunan Town, Mt. Yamabushiyama, (37.0010°N, 138.5936°E), 341 m elev., 12 November 2020, mature on 01 December 2020, T. Nagai leg., gaps between stones near mountain stream, (MNSK-Nes001) • 1♀, same locality, collection date, and environment, T. Nagai leg. (MNSK-Nes002) • 1♂, 2♀, same locality, collection date, and environment, (36.9998°N, 138.5904°E), 306 m elev., T. Nagai leg. (MNHH).

##### Other materials examined.

• 2♀, Amamizu Park (37.0508°N, 138.5854°E), 446 m elev., 03 November 2020, T. Nagai leg., gaps between stones at park, (TNPC) • 1♀, Mt. Yamabushiyama (37.0161°N, 138.5817°E), 651 m elev., 12 December 2020, T. Nagai leg., gaps between stones near mountain stream, (TNPC) • 1♀, same locality and environment, 22 March 2021, (36.9998°N, 138.5904°E), 306 m elev., T. Nagai leg. (TNPC).

##### Etymology.

The specific name is derived from the type locality, Mount Yamabushiyama (山伏山). It is treated as a noun in apposition.

##### Diagnosis.

**Figure 5.**
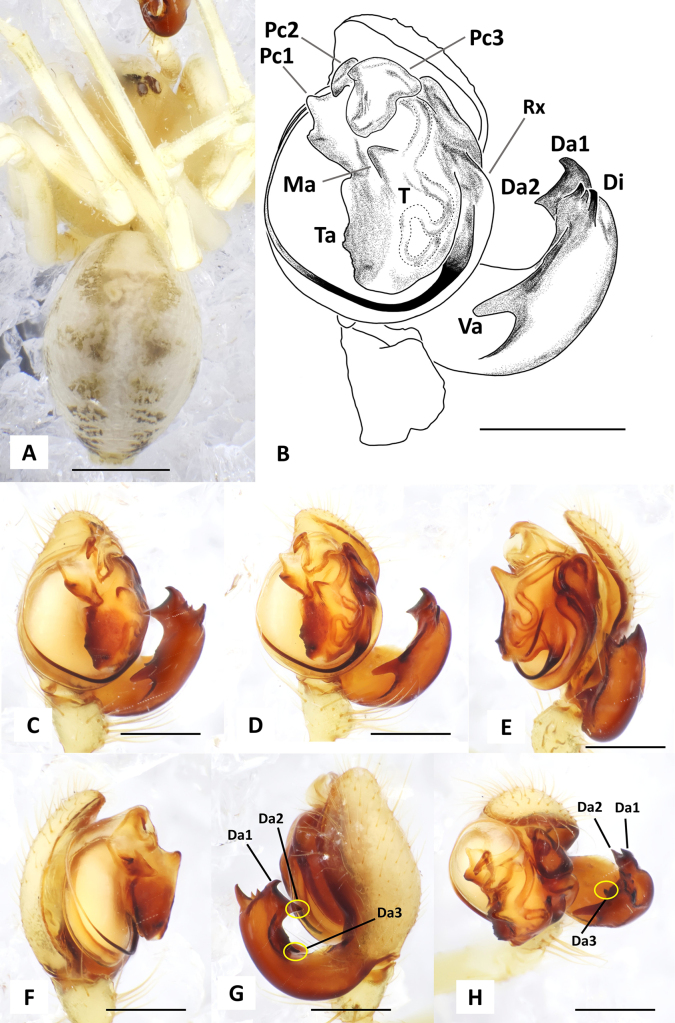
*Nesticus echigonus*, male habitus and genitalia of holotype. **A**. Habitus dorsal; **B**. Genital sketch, left palp, ventral; **C**. Left palp, ventral; **D**. Same, ventro-retrolateral; **E**. Same, distal; **F**. Same, medial; **G**. Same, dorso-retrolateral; **H**. Posterior. Abbreviations: Da1–3 = dorsal apophyses of paracymbium, Di = distal apophysis of paracymbium, Ma = median apophysis, Pc1–3 = processes of conductor complex, Rx = radix, T = tegulum, Ta = tegular apophysis, Va = ventral apophysis of paracymbium. Scale bars: 1.0 mm (**A**); 0.5 mm (**B–H**).

*Nesticus yamabushi* sp. nov. closely resembles *Nesticus gondai* Yaginuma, 1979 and *Nesticus echigonus* Yaginuma, 1986 in the morphology of the genitalia. Differences between the new species and the above two congeneric species are as follows. Male of the new species can be distinguished from males of the latter two species by the following combination of characters: a massive, conical median apophysis (Ma), approximately two times longer than its width at the base, and ending with a pointed tip (vs smaller, with length approximately equal to its width at the base in both other species, but also rectangular in *N. gondai*); a tegular apophysis (Ta) with a triangular projection and several small denticles at its base (vs a single small projection lacking denticles in *N. gondai*, or lacking any clear projection in *N. echigonus*); the presence of a 4^th^ process (Pc4) of the conductor complex (vs absent). Additionally, the paracymbium of the new species shows several differences in its projections including a distal process (Di) very long and pointed (vs very short in the other species). (cf. Fig. 3A–G vs Figs 5B–H, 8B–H, 8Yaginuma 1979: figs 2–1, 2–4, and Yaginuma 1986: figs 6, 7).

**Figure 6.**
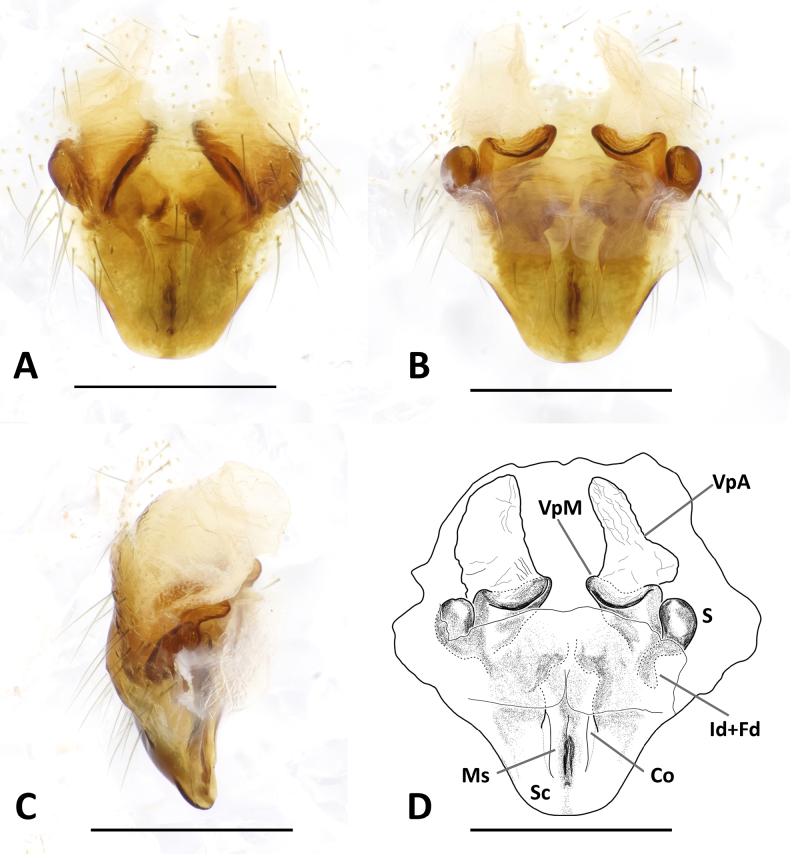
*Nesticus echigonus* female genitalia of specimen NSMT-Ar 27112. **A**. Epigyne, ventral; **B**. Vulva, dorsal; **C**. Epigyne, lateral; **D**. Genital sketch, vulva, dorsal. Abbreviations: Co = copulatory opening, Id+Fd = insemination + fertilization ducts, Ms = median septum, S = spermatheca, Sc = scape, VpA = anterior vulval pocket, VpM = medial vulval pocket. Scale bars: 0.5 mm.

Females of the new species can be distinguished from females of *N. echigonus* and *N. gondai* by the following combination of characters: scape (Sc) trapezoidal (vs more triangular); with a wide distal end concave posteriorly (vs flat or convex); medial vulval pocket (VpM) plate-like and extended anteriorly (vs posteriorly convex in *N. echigonus* and medially extending in *N. gondai*) (cf. Fig. 4A–D vs Figs 6A–D, 9A–D, Yaginuma 1979: figs 2–2, 2–3, Yaginuma 1986: figs 11, 12).

##### Description of male (holotype).

Total length 3.37. Prosoma 1.74 long, 1.64 wide. Habitus as in Fig. 2A. Carapace yellowish brown, darker along midline and edge. Eye diameter: AME 0.08, ALE 0.12, PME 0.12, PLE 0.12. Eye interdistances: AME−ALE 0.05, ALE−PLE 0.00, PME−PLE 0.03. Clypeus 0.28 long. Chelicerae yellowish brown, maxillae whitish yellow, white near pit; labium yellowish grey. Sternum yellowish grey, with dark-coloured edging. Legs yellowish brown, with dark bands. Legs measurements: I 14.53 (3.85, 0.84, 4.10, 4.12, 1.61), II 10.88 (3.07, 0.80, 2.84, 2.91, 1.26), III 8.09 (2.48, 0.63, 1.95, 2.04, 0.98), IV 11.01 (3.35, 0.74, 2.87, 2.90, 1.15). Leg formula: I, IV, II, III. Dorsal opisthosoma whitish yellow, with black paired marks; ventral opisthosoma anteriorly yellowish grey, posteriorly whitish yellow, with a pair of darkish stripes, greyish yellow around spinnerets. PLS yellowish grey; others whitish yellow.

Palps as in Fig. 3A–G. Cymbium (C) oval, with sparse setae. Sparse setae on dorsal side of paracymbium (P) and tibia. Paracymbium with 1 distal (Di), 2 dorsal (Da1–2) and 1 ventral (Va) apophyses: Di sharp and long, curved medially with a 90° angle, ending pointed; Da1 shorter than Di and curved anteriorly, tough, protruding anteriorly, ending sharp; Da2 short and thick, pointed, headed dorsally; Va bulky, protruding medially, ending rounded. Embolus (E) black and filiform, starting at approximately 5-o’clock position on radix (Rx). Median apophysis (Ma) conical, with pointed tip, strongly protruding dorso-retrolaterally, approximately 2 times longer than its width at base. Tegular apophysis (Ta) strongly sclerotized, triangular, with several saw-like denticles on prolateral edge of radix. Conductor complex with 4 processes (Pc1−4): (Pc1) plate-like, with single sclerotized and spatula-like projection on posterior side; Pc2 anterior to Pc1, sickle-shaped, strongly sclerotized, ending pointed; Pc3 ventral to Pc2, shaped as a translucent plate with margin curving ventrally, bearing a dorsal projection extending counterclockwise and wrapping around part of E; Pc4 club-shaped, strongly sclerotized, protruding prolaterally from base of Pc1 under Ma.

##### Description of female (one of the paratypes).

Total length: 4.30. Carapace 1.91 long, 1.71 wide. Habitus as in Fig. 2B. Eye diameter: AME 0.10, ALE 0.18, PME 0.14, PLE 0.15. Eye interdistances: AME–ALE 0.04, ALE−PLE 0.00, PME–PLE 0.07. Clypeus 0.26 long. Legs measurements: I 13.40 (3.75, 0.91, 3.69, 3.55, 1.50), II 10.19 (2.97, 0.86, 2.55, 2.63, 1.19), III 7.59 (2.36, 0.74, 1.64, 1.89, 0.97), IV 10.66 (3.33, 0.87, 2.67, 2.66, 1.13). Leg formula: I, IV, II, III. Colouration and other somatic features as in male.

Epigyne and vulva as in Fig. 4A–D. Scape (Sc) trapezoidal, with concave distal end, slightly bent dorsally when observed laterally. Sclerotized furrows extending from distal end of Sc to copulatory openings for a distance subequal to width of Sc before invaginating. Copulatory opening (Co) approximately at centre of Sc. Insemination and fertilization ducts (Id+Fd) extending anteriorly, reaching spermatheca (S) after turning laterally; medial vulval pocket (VpM) plate-like, extended anteriorly; anterior vulval pocket (VpA) wide, sack-like, translucent, and wrinkled, extending from anterior side of VpM. Spermatheca (S), small, rounded, located at base of VpM.

##### Size variation.

Male (*n* = 3): total length: 3.56–4.59; carapace 1.74–2.01 long, 1.64–1.84 wide; female (*n* = 5): total length: 3.96–5.52, carapace 1.80–1.94 long, 1.61–1.77 wide.

##### Distribution.

Known only from the surroundings of Mt. Yamabushiyama, in Niigata Prefecture, Japan (Fig. 10).

#### Nesticus echigonus

Taxon classificationAnimaliaAraneaeNesticidae

Yaginuma, 1986

2B808D01-2631-5AD2-BD30-5C16B7D705D6

**Figure 7.**
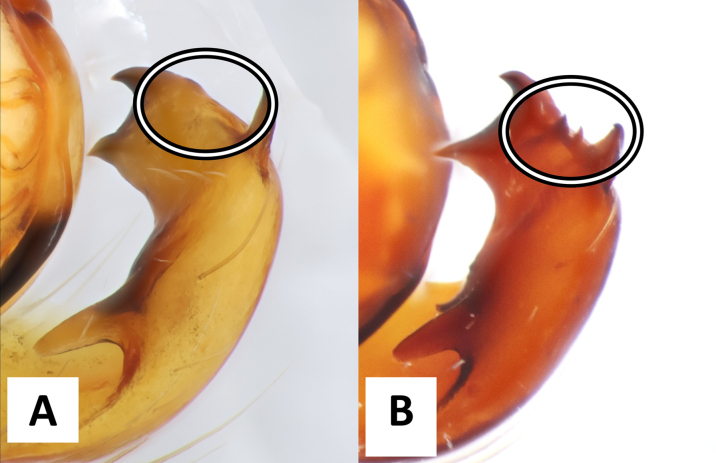
Morphological variation in the paracymbium of the male palp in *Nesticus echigonus*. **A**. Paracymbium of the specimen NSMT-Ar 27112 lacking additional denticles (white circle); **B**. Paracymbium of the holotype with denticles (white circle).

Figs 2C, 2D, 5A–H, 6A–D, 7A, 7B

Nesticus echigonus Yaginuma, 1986: 27, figs 6–12, (original description).
Nesticus echigonus —Kamura and Irie 2009: 349, figs 34, 35.

##### Japanese name.

Echigo-Horahimegumo エチゴホラヒメグモ.

##### Materials examined.

**Japan**: Honshu Island, Niigata Pref.: • 1♂ (holotype), Kitauonuma-gun, Irihirose-mura (now Uonuma city), Mt. Asakusadake, 1010 m elev., 5 August 1984, S.-I. Uéno leg. (NSMT-Ar 1914) • 1♂, 1♀ (paratypes), same locality and date (NSMT-Ar 1915 and Ar 1917) • 1♂, same locality (37.3561°N, 139.2204°E), 937 m elev., juvenile specimen, collected on 03 July 2021, matured in laboratory on 15 July 2021, T. Nagai leg., gaps between stones near mountain stream (NSMT-Ar 27111) • 1♀, same locality, 30 October 2023, T. Nagai leg., gaps between stones near mountain stream (NSMT-Ar 27112) • 1♀, same locality, date, and environment, T. Nagai leg. (TNPC).

##### Diagnosis.

**Figure 8.**
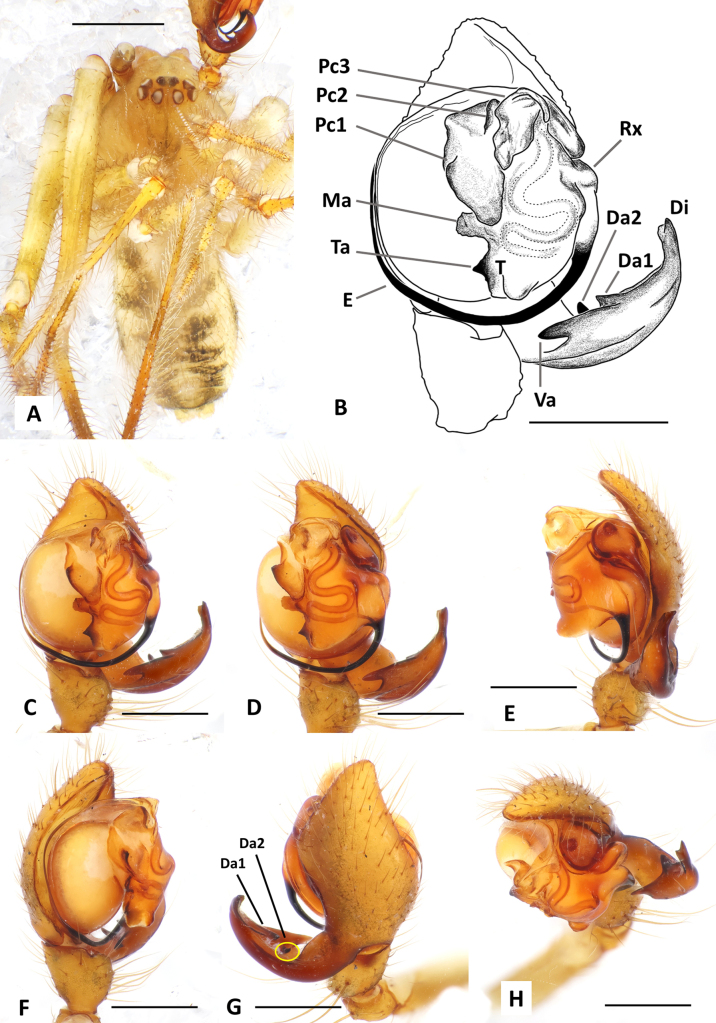
*Nesticus gondai*, male habitus and genitalia of holotype. **A**. Habitus, dorsal; **B**. Genital sketch, left palp, ventral; **C**. Left palp, ventral; **D**. Same, ventro-retrolateral; **E**. Same, distal; **F**. Same, medial; **G**. Same, dorso-retrolateral; **H**. Same, posterior. Abbreviations: Da1–2 = dorsal apophyses of paracymbium, Di = distal apophysis of paracymbium, E = embolus, Ma = median apophysis, Pc1–3 = processes of conductor complex, Rx = radix, T = tegulum, Ta = tegular apophysis, Va = ventral apophysis of paracymbium. Scale bars: 1.0 mm (**A**), 0.5 mm (**B–H**).

Male of *N. echigonus* can be distinguished from males of *N. gondai* by the following combination of characters: median apophysis (Ma) conical, ending sharp (vs rectangular, ending blunt); tegular apophysis (Ta) scarcely developed forming only small, short bumps (vs well developed, triangular). Additionally, the paracymbium (P) shows several differences in its projections including two curved dorsal apophyses (Da1–2) situated near the distal portion of P (vs straight, located at the middle) and Di shaped as a tapering plate-like structure (vs clavate and bearing two apices) (cf. Fig. 5B–H, Yaginuma 1986: figs 5–7 vs Fig. 8B–H, Yaginuma 1979: figs 2–1, 2–4).

**Figure 9.**
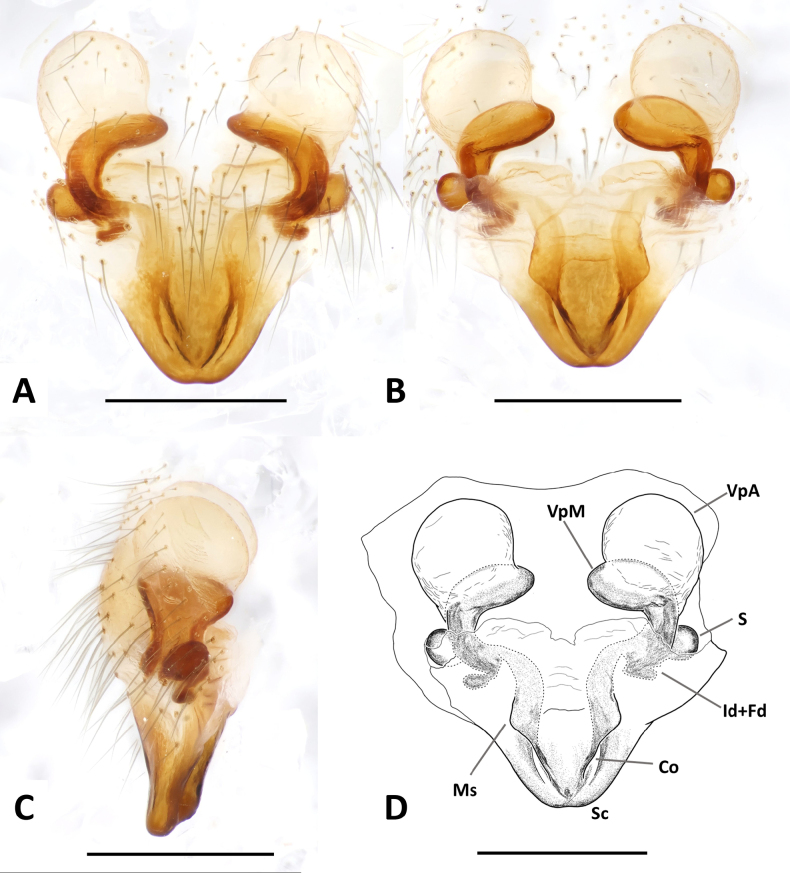
*Nesticus gondai* female genitalia of the specimen NSMT-Ar 27114. **A**. Epigyne, ventral; **B**. Vulva, dorsal; **C**. Epigyne, lateral; **D**. Genital sketch, vulva, dorsal. Abbreviations: Co = copulatory opening, Id+Fd = insemination + fertilization ducts, Ms = median septum, S = spermatheca, Sc = scape, VpA = anterior vulval pocket, VpM = medial vulval pocket. Scale bars: 0.5 mm.

Female of *N. echigonus* can be distinguished from female of *N. gondai* by the following combination of characters: the scape (Sc) is flattened at the distal end (vs tapering); a median septum (Ms) on dorsal side of Sc with parallel borders (vs V-shaped, cf. Fig. 6A–D, Yaginuma 1986: figs 11, 12 vs Fig. 9A–D, Yaginuma 1979: figs 2–2, 2–3).

For comparisons with *N. yamabushi* sp. nov., see the diagnosis of that species.

##### Redescription of male (holotype).

Total length 4.27. Prosoma 1.95 long, 1.79 wide. Habitus as in Fig. 5A; habitus of a freshly collected individual as in Fig. 2C. Carapace light yellowish brown, slightly darker along midline and edge. Eye diameter: AME 0.07, ALE 0.13, PME 0.16, PLE 0.13. Eye interdistances: AME−ALE 0.03, ALE−PLE 0.00, PME−PLE 0.03. Clypeus 0.25 long. Chelicerae whitish yellow; maxillae whitish yellow, white near pit; labium whitish yellow. Sternum yellowish grey, with dark coloured edging. Legs whitish yellow, with indistinct dark bands. Legs measurements: I 14.49 (3.94, 0.94, 4.09, 4.04, 1.47), II 11.18 (3.17, 0.86, 2.88, 3.09, 1.18), III 8.30 (2.55, 0.69, 1.89, 2.22, 0.95), IV 11.07 (3.36, 0.70, 2.98, 2.98, 1.05). Leg formula: I, II, IV, III. Dorsal opisthosoma greyish yellow, with black paired marks; ventral opisthosoma anteriorly greyish yellow, posteriorly greyish yellow, darker around spinnerets. Spinnerets whitish yellow.

Palps as in Fig. 5B−H. Cymbium (C) oval, with sparse setae. Sparse setae on dorsal side of paracymbium (P) and tibia. Paracymbium (P) with 1 distal (Di), 3 dorsal (Da1–3), and 1 ventral (Va) apophyses: Di small, thorn-like, antero-dorsally protruding, Da1−3 short and pointed, Da1 and Da2 curved, dorso-medially protruding at distal part of P; Da3 medially protruding; Va bulky, medially protruding, ending with a rounded tip. Two additional small denticles between Da1 and Di (but see remarks on variation). Embolus (E) black and filiform, starting at approximately 5-o’clock position on radix (Rx). Median apophysis (Ma) conical, protruding medially, approximately as long as its width at base. Tegular apophysis (Ta) only slightly developed, shaped as tiny, sclerotized bumps. Conductor complex with 3 processes (Pc1−3): Pc1 plate-like, with single sclerotized projection on posterior side; Pc2 anterior to Pc1, sickle-shaped, ending pointed; Pc3 ventral to Pc2, shaped as a translucent plate with its margin curving ventrally, bearing a dorsal projection extending counterclockwise and wrapping around a part of E.

##### Redescription of female (one voucher specimen).

Total length: 3.86. Carapace 1.86 long, 1.64 wide. Habitus as in Fig. 2D. Eye diameter: AME 0.09, ALE 0.15, PME 0.15, PLE 0.15. Eye interdistances: AME−ALE 0.03, ALE−PLE 0.00, PME−PLE 0.04. Clypeus 0.23 long. Legs measurements: I 12.41 (3.52, 0.75, 3.41, 3.22, 1.50), II 9.90 (2.90, 0.82, 2.50, 2.51, 1.17), III 7.07 (2.24, 0.68, 1.50, 1.73, 0.93), IV 9.86 (3.16, 0.74, 2.82, 2.76, 1.19). Leg formula: I, II, IV, III. Colouration and other somatic features as in male.

Epigyne and vulva as in Fig. 6A−D. Scape (Sc) tongue-like, with curved distal end slightly bent dorsally when observed laterally. Median septum (Ms) on dorsal side of Sc extending along the median line with parallel borders. Copulatory openings (Co) approximately at center of Sc. Insemination and fertilization ducts (Id+Fd) anteriorly extending, reaching spermatheca (S) after laterally turning; medial vulval pocket (VpM) sclerotized, posteriorly convex; anterior vulval pocket (VpA) wide, sack-like, translucent and wrinkled, extending from anterior side of VpM. Spermatheca (S), small, rounded, located at base of VpM.

##### Size variation.

Male (*n* = 2): total length: 3.67–4.27; carapace 1.88–1.95 long, 1.78–1.79 wide; female (*n* = 3): total length: 3.48–5.12, carapace 1.64–2.13 long, 1.39–1.97 wide.

##### Remarks on variation.

The presence of small denticles on the distal end of the paracymbium of the male palp, between Da1 and Di, is variable, as some individuals lack one or both these apophyses (cf. Fig. 7A vs Fig. 7B vs Yaginuma 1986: figs 7−10).

##### Distribution.

**Figure 10.**
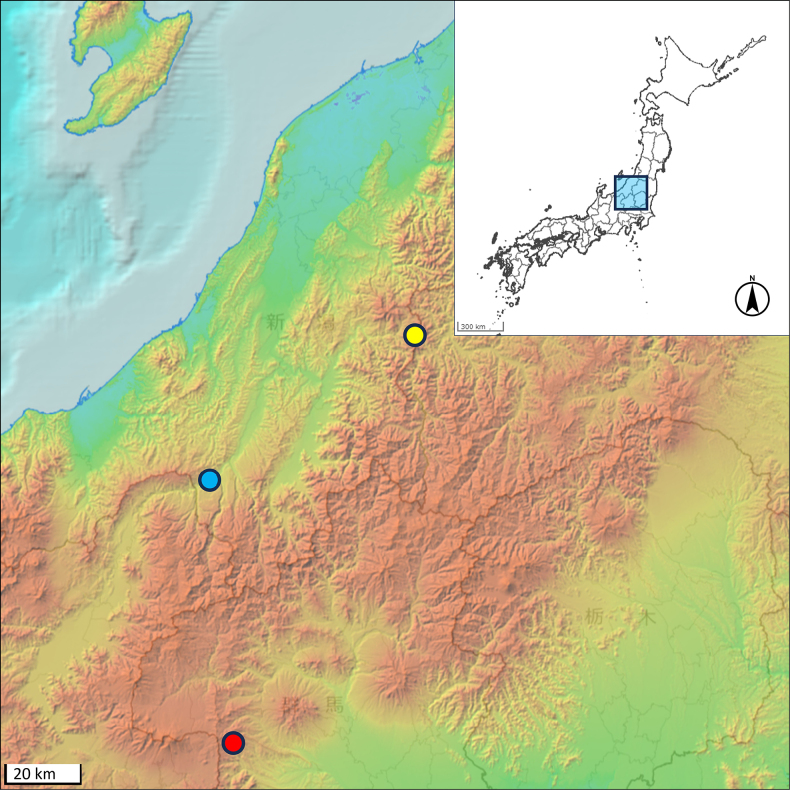
Map plotting the collection sites of *Nesticus echigonus* (yellow dot), *N. gondai* (red dot), and *N. yamabushi* sp. nov. (blue dot). The white map in the upper right corner indicates mainland Japan, the light blue square indicates the enlarged area. Map created based on the Colour-coded Elevation Map and the Blank Map provided by the Geospatial Information Authority of Japan (https://maps.gsi.go.jp).

Known only from the surrounding area of Mt. Asakusadake, in Niigata Prefecture, Japan (Fig. 10).

#### Nesticus gondai

Taxon classificationAnimaliaAraneaeNesticidae

Yaginuma, 1979

1C47B9B9-F59E-503E-B538-E79515A7EB5D

Figs 2E, 2F, 8A–H, 9A–D

Nesticus gondai Yaginuma, 1979: 263, pl. 2, figs 1–4 (original description).
Cyclocarcina gondai —Lehtinen and Saaristo 1980: 59 (from Nesticus).
Nesticus gondai —Kamura and Irie 2009: 345, figs 15, 16

##### Japanese name.

Kirizumi-Horahimegumo キリズミホラヒメグモ.

##### Materials examined.

Japan: Honshu Island, Gunma Pref.: • 1♂ (holotype), Usui-gun, Kirizumi (now Annaka City, Matsuida town, Sakamoto), 27 July 1970, S. Gonda leg. (OMNH-Ar Type-86) • 1♀ (paratype), same locality and data (OMNH) • 1♂, Annaka City, Matsuida town, Sakamoto, nearby Kirizumi dam lake, (36.36418°N, 138.7043°E), 569 m elev., 27 December 2020, T. Nagai leg., inside a culvert (NSMT-Ar 27113) • 1♀, same locality, (36.3685°N, 138.6989°E), 660 m elev., 28 March 2026, T. Nagai leg., inside a culvert (NSMT-Ar 27114) • 1♀, same locality, date, and environment, T. Nagai leg. (TNPC).

##### Diagnosis.

See the diagnosis of *N. yamabushi* sp. nov. and *N. echigonus*.

##### Redescription of male (holotype).

Total length 3.97. Prosoma 2.00 long, 1.77 wide. Habitus as in Fig. 8A, habitus of a freshly collected individual as in Fig. 2E. Carapace light yellowish brown, darker along midline and edge. Eye diameter: AME 0.11, ALE 0.14, PME 0.15, PLE 0.13. Eye interdistances: AME−ALE 0.04, ALE−PLE 0.00, PME−PLE 0.04. Clypeus 0.41 long. Chelicerae whitish yellow, maxillae whitish yellow, white near pit, labium yellowish grey. Sternum yellowish grey, with dark edging. Legs yellowish brown, with indistinct dark bands. Legs measurements: I 15.39 (4.13, 0.94, 4.30, 4.37, 1.63), II 11.97 (3.41, 0.84, 3.08, 3.33, 1.31), III 8.88 (2.72, 0.69, 2.00, 2.45, 1.02), IV 12.31 (3.76, 0.80, 3.13, 3.41, 1.20). Leg formula: I, IV, II, III. Dorsal opisthosoma whitish yellow, with paired black marks; ventral opisthosoma anteriorly yellowish grey, posteriorly whitish yellow, with darkish U-shaped band, greyish yellow around spinnerets. ALS whitish yellow, others yellowish grey.

Palps as in Fig. 8B–H. Cymbium (C) oval, with sparse setae. Sparse setae on dorsal side of paracymbium (P) and tibia. Paracymbium with 1 distal (Di), 2 dorsal (Da1–2), and 1 ventral (Va) apophyses: Di extending antero-dorsally, clavate, with flattened, elongated tip with two prongs; Da1 and Da2 on dorsal-medial side of the P, both short and sharp, Da1 connected to Di by a furrow; Va bulky, protruding medially, tapering and flattened toward the tip. Embolus (E) black and filiform, starting at approximately 5-o’clock position on radix (Rx). Median apophysis (Ma) rectangular, medially protruding, with blunt end. Tegular apophysis (Ta) spine-like, strongly sclerotized. Conductor complex with 3 processes (Pc1−3): Pc1 plate-like, with single sclerotized projection on posterior side; Pc2 anterior to Pc1, sickle-shaped with pointed tip; Pc3 ventral to Pc2, shaped as a translucent plate with its margin curving ventrally, bearing a dorsal projection extending counterclockwise and wrapping around a part of E.

##### Redescription of female (one voucher specimen).

Total length: 4.25. Carapace 2.04 long, 1.80 wide. Habitus as in Fig. 2F. Eye diameter: AME 0.11, ALE 0.17, PME 0.14, PLE 0.15. Eye interdistances: AME–ALE 0.06, ALE−PLE 0.00, PME–PLE 0.06. Clypeus 0.33 long. Legs measurements: I 14.30 (4.04, 0.96, 3.96, 3.76, 1.58), II 11.25 (2.65, 0.75, 2.87, 2.81, 1.25), III 9.32 (2.65, 0.75, 1.87, 2.10, 1.21), IV 11.54 (3.73, 0.87, 2.93, 2.80, 1.21). Leg formula: I, IV, II, III. Colouration and other somatic features as in male.

Epigyne and vulva as in Fig. 9A–D. Scape (Sc) subtriangular, distally tapering, with flat distal end slightly ventrally bent when observed laterally. Median septum (Ms) on dorsal side of Sc V-shaped, distally protruding, extending from tip of Sc to copulatory openings (Co) for a distance subequal to width of Sc then following curvature of copulatory opening (Co). Copulatory opening (Co) approximately at centre of Sc. Insemination and fertilization ducts (Id+Fd) extending anteriorly, reaching spermatheca (S) after turning laterally; medial vulval pocket (VpM) curved and rounded, extending medially; anterior vulval pocket (VpA) round, translucent and wrinkled, extending from anterior side of VpM. Spermatheca (S) small, rounded, located at base of VpM.

##### Size variation.

Male (*n* = 2): total length: 3.97–4.21; carapace 1.95–2.00 long, 1.75–1.78 wide; female (*n* = 3): total length: 3.66–4.25, carapace 1.54–2.04 long, 1.48–1.80 wide.

##### Distribution.

Known only from the surroundings of Kirizumi-onsen spring, Annaka City, Gunma Prefecture, Japan (Fig. 10).

##### Molecular analysis.

**Figure 11.**
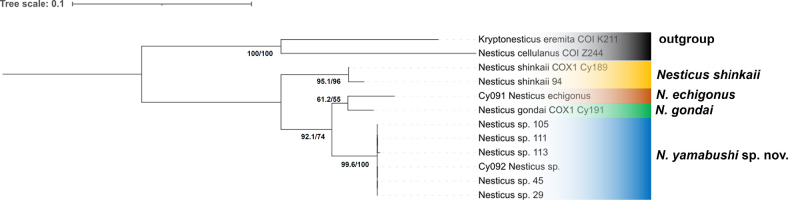
A phylogenetic tree based on mitochondrial *COI* sequences that was constructed using maximum-likelihood analyses using IQ-TREE v. 3. Branch lengths are scaled in relation to the number of substitutions per site. Node support values are indicated as Bootstrap Value/SH-aLRT.

In our phylogenetic tree (Fig. 11), *Nesticus yamabushi* sp. nov. was recovered as monophyletic, forming a distinct clade (Bootstrap Value/SH-aLRT: 99.6/100), clearly separated from its close relatives, *N. gondai* and *N. echigonus* (92.1/74) as its putative sister group. All these clades exhibited long basal branches, supporting their separation.

In the analysis of genetic distances (Table 2), the divergence between *N. yamabushi* sp. nov. and *N. gondai* was 3.5%. The genetic distances between *N. yamabushi* sp. nov. and *N. echigonus* ranged from 4.4% to 5.2%, while the distance between *N. echigonus* and *N. gondai* was 4.3%. These values provide further evidence that all three species treated in this study are genetically differentiated from one another. The maximum intraspecific genetic distance within *N. yamabushi* sp. nov. was only 0.2%.

**Table 2.** Genetic *p*-distance among *Nesticus yamabushi* sp. nov. and other known species. The values are based on the *COI* partial sequence discussed in the text.

		**1**	**2**	**3**	**4**	**5**	**6**	**7**	**8**	**9**	**10**	**11**
1	29 *Nesticus yamabushi***sp. nov**.											
2	45 *Nesticus yamabushi***sp. nov**.	0.000										
3	105 *Nesticus yamabushi***sp. nov**.	0.000	0.000									
4	111 *Nesticus yamabushi***sp. nov**.	0.000	0.000	0.000								
5	113 *Nesticus yamabushi***sp. nov**.	0.002	0.002	0.000	0.000							
6	Cy092 *Nesticus yamabushi***sp. nov**.	0.000	0.000	0.000	0.000	0.002						
7	Cy091 *Nesticus echigonus*	0.034	0.034	0.034	0.035	0.035	0.033					
8	Cy191 *Nesticus gondai*	0.050	0.050	0.050	0.051	0.044	0.049	0.034				
9	94 *Nesticus shinkaii*	0.075	0.075	0.075	0.075	0.073	0.075	0.074	0.078			
10	Cy189 *Nesticus shinkaii*	0.064	0.064	0.064	0.066	0.066	0.064	0.068	0.072	0.009		
11	Z244 *Nesticus cellulanus*	0.150	0.150	0.151	0.151	0.162	0.150	0.150	0.138	0.142	0.153	
12	K211 *Kryptonesticus eremita*	0.136	0.136	0.136	0.139	0.144	0.135	0.137	0.136	0.145	0.150	0.114

## Discussion and conclusions

In this study, we conducted an integrative analysis comparing *Nesticus* species from the same geographic area in central Japan. Our results demonstrate that the three closely related species present in the Kanto-Koshinetsu region, *N. yamabushi* sp. nov., *N. echigonus*, and *N. gondai*, are clearly distinguishable based on both morphological and molecular data. We provide evidence supporting the validity of *Nesticus yamabushi* sp. nov. as a distinct species described herein and clarify the taxonomic and systematic status of *N. gondai* and *N. echigonus*.

In particular, our phylogenetic analysis based on the mitochondrial *COI* gene sequences recovered all three species as well-supported monophyletic clades, distinct from each other. The interspecific genetic distances between them align with the 3–4% divergence threshold typically recognized for interspecific differentiation in spiders (Barrett and Hebert 2005), further supporting their status as distinct species.

Morphologically, all three species exhibit stable diagnostic characters in both sexes. The updated morphological accounts provided here for *N. gondai* and *N. echigonus* address the general lack of information available from their original descriptions and confirm their limited distribution. Currently, all these species are known exclusively from the vicinity of their respective type localities in Gunma and Niigata Prefectures in central Japan. Such a restricted distribution is consistent with the high level of endemism often found in Japanese *Nesticus* species. Additionally, the discovery of a new locally endemic species further suggests that the diversity of the Japanese nesticid fauna remains underestimated even at a regional scale. It also highlights the effectiveness of integrative taxonomic approaches for clarifying species boundaries among closely related taxa and revealing the hidden diversity within hyper-diverse Japanese spider genera such as *Nesticus*.

## Supplementary Material

XML Treatment for Nesticus

XML Treatment for Nesticus yamabushi

XML Treatment for Nesticus echigonus

XML Treatment for Nesticus gondai

## Additional information

### Artificial Intelligence (AI) use

The authors accept full responsibility for the content of the manuscript, including the disclosure of any use of AI.

Regarding the use of AI in the preparation of this manuscript, the authors declare the following: Claude, Gemini.

**Used for**: Language, style and writing, Literature discovery and brainstorming.

English language editing and preliminary literature search were assisted by the AI tools Gemini (Google) and Claude (Anthropic).

### Author ORCIDs

T. Nagai https://orcid.org/0009-0009-8327-2987

F. Ballarin https://orcid.org/0000-0003-1417-2519

K. Eguchi https://orcid.org/0000-0002-1054-1295

### Data availability

All of the data that support the findings of this study are available in the main text.
